# Aging-Invariant Sheep Face Recognition Through Feature Decoupling

**DOI:** 10.3390/ani15152299

**Published:** 2025-08-06

**Authors:** Suhui Liu, Chuanzhong Xuan, Zhaohui Tang, Guangpu Wang, Xinyu Gao, Zhipan Wang

**Affiliations:** 1 College of Electromechanical Engineering, Inner Mongolia Agricultural University, Hohhot 010018, China; lsh@emails.imau.edu.cn (S.L.); tangzhaohui@imau.edu.cn (Z.T.); 19528240518@163.com (G.W.); gaoxinyu820820@163.com (X.G.); 2 Inner Mongolia Engineering Research Center for Intelligent Facilities in Prataculture and Livestock Breeding, Hohhot 010018, China; 3 Shenzhen Key Laboratory of Intelligent licrosatellite Constellation, Shenzhen Campus of Sun Yat-sen University, No. 66, Gongchang Road, Guangming District, Shenzhen 518107, China; wangzhp25@mail2.sysu.edu.cn; 4 School of Aeronautical Engineering, Changsha University of Science & Technology, Changsha 410004, China

**Keywords:** agricultural engineering, biometric recognition, sheep facial features, feature decoupling, deep learning

## Abstract

Precise recognition of individual ovine specimens plays a pivotal role in implementing smart agricultural platforms and optimizing herd management systems. With the development of deep learning technology, sheep face recognition provides an efficient and contactless solution for individual sheep identification. However, with the growth of sheep, their facial features keep changing, which poses challenges for existing sheep face recognition models to maintain accuracy across the dynamic changes in facial features over time, making it difficult to meet practical needs. To address this limitation, we propose the lifelong biometric learning of the sheep face network (LBL-SheepNet), a feature decoupling network designed for continuous adaptation to ovine facial changes, and constructed a dataset of 31,200 images from 55 sheep tracked monthly from 1 to 12 months of age. The LBL-SheepNet model addresses dynamic variations in facial features during sheep growth through a multi-module architectural framework. Firstly, a Squeeze-and-Excitation (SE) module enhances discriminative feature representation through adaptive channel-wise recalibration. Then, a nonlinear feature decoupling module employs a hybrid channel-batch attention mechanism to separate age-related features from identity-specific characteristics. Finally, a correlation analysis module utilizes adversarial learning to suppress age-biased feature interference, ensuring focus on age-invariant identifiers. Experimental results demonstrate that LBL-SheepNet achieves 95.5% identification accuracy and 95.3% average precision on the sheep face dataset. This study introduces a lifelong biometric learning (LBL) mechanism to mitigate recognition accuracy degradation caused by dynamic facial feature variations in growing sheep. By designing a feature decoupling network integrated with adversarial age-invariant learning, the proposed method addresses the performance limitations of existing models in long-term individual identification.

## Simple Summary

To tackle the issue of maintaining accuracy in sheep face recognition across different growth stages, we constructed a dataset of 31,200 images from 55 sheep tracked monthly from 1 to 12 months of age. We proposed the LBL-SheepNet framework, which includes a Squeeze-and-Excitation (SE) module for enhancing feature representation, a nonlinear feature decoupling module for separating age-related features from identity-specific ones, and a correlation analysis module with adversarial learning to reduce age-biased interference. The framework achieved 95.5% identification accuracy and 95.3% average precision.

## 1. Introduction

In large-scale livestock farms, livestock identification plays a crucial role in intelligent animal husbandry and modernized management, which helps improve farming efficiency and ensures food safety and traceability [1,2]. Sheep are timid and sensitive, and the traditional identification method of ear tagging suffers from problems such as pain, stress, and tag loss [3,4]. Utilizing non-contact methods such as DNA recognition, facial recognition for sheep, or iris scanning can effectively prevent excessive contact with the sheep, thereby avoiding potential stress or agitation [5,6,7]. However, the acquisition of iris biometrics and genetic material necessitates specialized instrumentation with substantial cost barriers, coupled with computationally intensive processing workflows that extend operational timelines. In contrast, facial recognition offers clear advantages—convenience, non-invasiveness, and user acceptance—over DNA-based fingerprinting and iris scanning for sheep identification [8,9]. Considering the growth cycle of sheep, which commonly reaches the standard for marketing within a year, there is a pronounced transformation in their facial features from birth to adulthood. These rapid physiological changes pose a significant challenge to sheep face recognition technology, as conventional models may struggle to accommodate such dynamic fluctuations. The study of lifelong biometric learning for sheep face recognition is crucial for enhancing the adaptability and accuracy of recognition techniques. Developing sophisticated models capable of accurately identifying sheep’s facial features throughout their lifespan can significantly improve the efficiency of sheep management.

In recent years, scholars have proposed some methods for sheep face identification. In facial recognition research, Yang et al. [10] put forward a triple interpolation feature technique for aligning human and sheep faces, which led to enhanced outcomes when applied to sheep face datasets. Salama et al. [11] designed a sheep face recognition system by merging deep learning with Bayesian optimization. Their approach leveraged the Bayesian algorithm to optimize the CNN automatically, attaining a recognition success rate of up to 98%. Wei et al. [12] developed a goat face detection and recognition technique grounded in deep learning, utilizing conventional feature extraction approaches as its foundation. Billah et al. [13] applied the YOLOv4 network to discern key goat facial attributes, such as the face, eyes, ears, and nose, achieving accuracy rates of 93%, 83%, 92%, and 85%, respectively. Zhang et al. [14] constructed a sheep face recognition model utilizing the Vision Transformer (ViT) architecture, with the goal of refining animal management practices and enhancing welfare. In a subsequent study, Zhang et al. [15] employed convolutional neural networks (CNNs) to create a lightweight model for sheep face recognition, addressing challenges in sheep identification and achieving high accuracy while being suitable for deployment on edge devices. Li et al. [16] introduced MobileViTFace, a balanced model for sheep face recognition that harnesses the power of Transformers to better capture fine-grained features while diminishing background noise, thereby improving the model’s ability to distinguish between different sheep faces. Later, Li et al. [17] proposed the Eblock, an efficient and swift foundational component used to develop the lightweight SheepFaceNet model, which successfully balanced speed and accuracy. These methodologies aimed to boost recognition efficiency by minimizing background interference, in turn uplifting recognition precision. Ning et al. [18] proposed a goat face recognition method based on improved YOLO v5s, which introduces the CARAFE up-sampling module to recover the facial details better and improve the model’s recognition accuracy of the individual faces of dairy goats, especially in the contactless individual recognition of dairy goats.

In summary, scholars have made significant progress in the field of sheep face recognition based on deep learning in recent years, and the research focuses on the lightness and robustness of the model, and has achieved certain results [19]. However, existing research has not yet addressed the problem of lifelong biometric learning for sheep face recognition. Sheep’s facial features change continuously with the growth process, and the initially deployed models are difficult to adapt to such dynamic changes, resulting in the degradation of recognition performance. Therefore, it is important to develop sheep face recognition models that can cope with this challenge. Inspired by the cross-growth face recognition network model [20,21,22], the study of lifelong biometric learning for sheep face recognition models can provide continuous authentication for sheep throughout their lifecycle, maintain stable model recognition, and effectively reduce the cost of secondary research investment. In addition, feature decoupling techniques show great potential in addressing the challenges of dynamically changing features for recognition tasks. For example, a recent study proposes a human activity recognition (HAR) model based on WiFi signals, which significantly improves the recognition performance by decoupling and restructuring gestures and identity features to generate virtual gesture samples for a new user domain [23]. This indicates that the feature decoupling technique can effectively separate dynamically changing features, thereby improving the adaptability and accuracy of the recognition system. Inspired by this, this study proposes a sheep face recognition network based on lifelong biometric learning. By decoupling sheep face features into growth features and identity features, this network can effectively address the limitations of traditional models in dealing with dynamic changes in sheep faces. Specifically, we introduce a feature decoupling module to reduce the interference of growth features on identity recognition through correlation analysis, thus improving the accuracy and robustness of sheep face recognition.

Sheep face recognition systems face significant challenges when dealing with lifelong biometric learning. Most existing datasets used for model training are based on images captured at a single time point, which fails to account for the dynamic changes in sheep facial features over their lifespan. In contrast, datasets that capture these changes require data collection over multiple time points, adding complexity to the acquisition process. Moreover, existing residual networks, such as ResNet, have shown limitations in extracting relevant features from sheep faces. Specifically, they struggle to distinguish between growth-related characteristics and identity-specific traits, diminishing the importance of growth factors in identity recognition. To address these challenges, this paper makes several contributions based on the latest advances in the field of sheep facial recognition:

(1) A facial dataset of sheep has been collected and built through long-term cooperation with a sheep farm, designed to comprehensively capture the dynamic changes in facial features over their lifespan for lifelong biometric learning, thereby providing a more robust basis for model training.
(2) An attention mechanism has been introduced into the ResNet residual structure to enhance the model’s ability to extract valid features from sheep faces throughout their lifespan, supporting effective lifelong biometric learning.
(3) A feature decoupling module has been proposed to effectively separate growth features from identity features. Through correlation analysis, the influence of age-related factors on identity features is reduced, thereby improving the accuracy and effectiveness of identity features in the recognition process.

## 2. Materials and Methods

### 2.1. Data Acquisition Device

**Figure 1:**
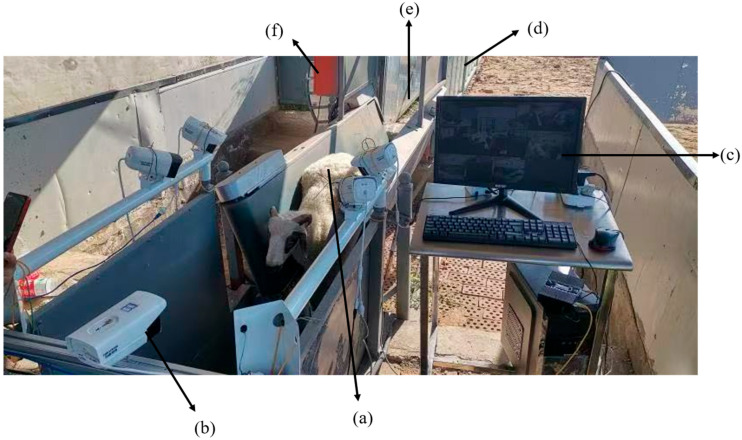
The overall structure of the sheep face image acquisition device: (a) a conveyor belt system; (b) an image camera system; (c) a host computer system; (d) a channel; (e) a gate; (f) an electric control box. Only the three highlighted cameras (left, frontal, right) are active for data collection; the two outer units serve as redundant backups.

The ovine facial-image acquisition device was deployed at a commercial livestock farm in Helinge’er County, Inner Mongolia Autonomous Region, China. The manufacturer of the ovine facial-image acquisition device is Beiqi Pharmaceutical Equipment Co., Ltd., located in Helinge’er County, Hohhot City, Inner Mongolia Autonomous Region, China. As illustrated in Figure 1, the system comprises six components: a belt conveyor, an image-capture array, a host computer, a passageway, a gate, and an electrical control box. The imaging array is equipped with Hikvision B13HV3-IA PoE cameras providing 3-megapixel resolution, a 4 mm focal length, and a manufacturer-rated maximum monitoring distance of 6 m; the actual working distance in this study is 0.9–1.1 m. The manufacturer of the Hikvision B13HV3-IA PoE cameras is Hikvision Digital Technology Co., Ltd., located in Binjiang District, Hangzhou City, Zhejiang Province, China. Sheep enter via the gate onto the conveyor, which is inclined at 75° with a tapered lower section and a wider upper section to prevent leg slippage. Upon reaching the imaging zone, the controller halts the belt for ≤30 s. Although five PoE cameras are installed for redundancy, only the three centrally aligned units (left, frontal, right) are activated during normal acquisition; the two outer cameras remain as hot-standby units. Captured images are streamed in real time to the host computer. Sheep enter via the gate onto the conveyor, which is inclined at 75° with a tapered lower section and a wider upper section to prevent leg slippage. Upon reaching the imaging zone, the controller halts the belt for ≤30 s. Although five PoE cameras are installed for redundancy, only the three centrally aligned units (left, frontal, right) are activated during normal acquisition; the two outer cameras remain as hot-standby units. Captured images are streamed in real time to the host computer.

To confirm that the imaging procedure had no adverse welfare effects, we continuously monitored heart-rate telemetry (Polar H10) across five consecutive weeks in ten sheep. The observed mean increase in only 6 ± 3% relative to baseline fell well below the 15% threshold for mild stress [24], demonstrating that the protocol had a negligible impact on animal welfare. The total hardware cost for the imaging tunnel, including five Hikvision B13HV3-IA cameras (approximately 60 USD each) and PoE switches, is approximately 400 USD, which local farms find acceptable.

In this study, we employed two distinct image acquisition methods to ensure the diversity and representativeness of our dataset. For younger and more manageable sheep, we utilized handheld cameras for image capture, offering flexibility in obtaining images under various environmental conditions. For older and less controllable sheep, we employed the specialized data acquisition device described above. By integrating these two acquisition approaches, we were able to construct a comprehensive dataset that spans all stages of growth, from rapid changes to more subtle and relatively invariant facial features.

### 2.2. Sheep Face Dataset

In this study, we collected the facial images of sheep at various stages of growth from juvenile to old age through the data acquisition equipment in Section 2.1 to validate the feasibility of the model. The data collection site was selected at the livestock farm in Helinger County which cooperates with our group. Collection began in October 2021 and continued for 15 months, yielding longitudinal facial images from 55 sheep, each covering ages 1–12 months. The collected images included different lighting conditions, different postures, and multiple shooting angles to fully capture the complexity of the sheep’s facial features during growth [25].

**Figure 2:**
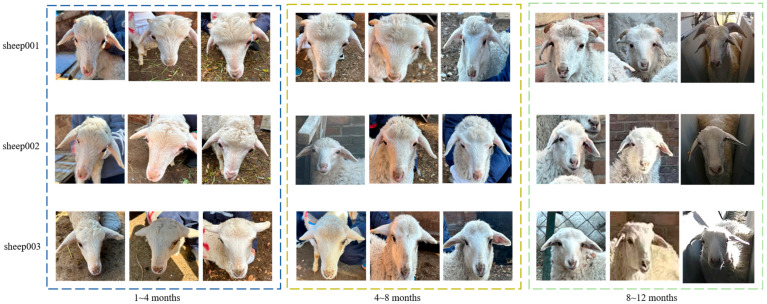
Example of sheep face image. The blue dotted box shows the facial images of sheep from 0 to 4 months of age, the yellow dotted box shows the facial images of sheep from 5 to 8 months of age, and the green dotted box shows the facial images of sheep from 9 to 12 months of age.

During post-processing, duplicate images were removed, and each photograph received a unique identifier corresponding to a hand-written code affixed to the left flank of every sheep; this code remained unchanged for the entire study. The sheep’s exact age (in months) at the moment of capture was subsequently entered manually into the acquisition software and embedded in the EXIF metadata of each image. An example of a sheep’s facial image is shown in Figure 2.

#### 2.2.1. Data Augmentation

Typically, the facial features of sheep undergo significant changes from 1 to 4 months of age with increasing growth stages. From 4 to 8 months of age, there are minor variations in facial characteristics. However, after 8 months of age, the facial features of sheep stabilize. Due to the vulnerability of newborn lambs, it is advisable to refrain from photographing them to avoid causing stress and potential health risks. Therefore, the use of flash and disturbances should be avoided during image capture. Therefore, only images of lambs aged 1 month and above were captured. Images of lambs aged 1 to 4 months were used for the training set; images of lambs aged 5 to 8 months were used for the validation set; and images of lambs aged 9 to 12 months were used for the test set. Therefore, each sheep in this study had at least three images captured at different growth stages throughout their lifespan. For each sheep, it was ensured that there were images available for the age ranges of 1~4 months, 5~8 months, and 9~12 months. During the collection process, efforts were made to capture images of each sheep from various angles, positions, and under different lighting conditions whenever possible. After removing highly similar images, a total of 8000 images were collected, comprising 5800 images for the age range of 1~4 months, 1000 images for the age range of 5~8 months, and 1000 images for the age range of 9~12 months. To avert the potential for facile learning and the resultant overfitting in the model training process, we employed a robust data augmentation technique to enhance the original images. This strategy involved fine-tuning the global luminance and contrast of the images, incorporating a regulated level of noise, and executing random rotational transformations on the facial images of the sheep. The original images in the test set were expanded from 5800 to 24,000 images, while those in the validation set were expanded from 1000 to 4000 images. Additionally, the original images in the test set were increased from 1000 to 4000 images. Data augmentation techniques have enriched our dataset’s diversity, allowing for a more nuanced apprehension of ovine facial features and augmenting the accuracy of the recognition model. The dataset summary is presented in Table 1.

#### 2.2.2. Normalization Processing

During the training process of deep neural networks, the output distribution of the activation functions in each layer changes with the increase in network depth, leading to internal covariate shifts. To address this issue, we have adopted batch normalization technology. This technology accelerates convergence and enhances the model’s generalization capability by normalizing the inputs to the layers, as shown in Equation (1).

(1) $${\hat{x}}^{\left(k\right)}=\frac{{\hat{x}}^{\left(k\right)}-\mu_{B}}{\sqrt{{\sigma^{2}}_{B}+\varepsilon }}$$

In the batch normalization process, ${\hat{x}}^{\left(k\right)}$ represents the value of the k-th feature within the current mini-batch, *μ_B_* denotes the mean of the mini-batch and *σ^2^_B_* signifies the variance of the mini-batch. The term *ε* is a tiny constant added to ensure numerical stability.

### 2.3. Lifelong Biometric Learning-Based Sheep Face Recognition Model

**Figure 3:**
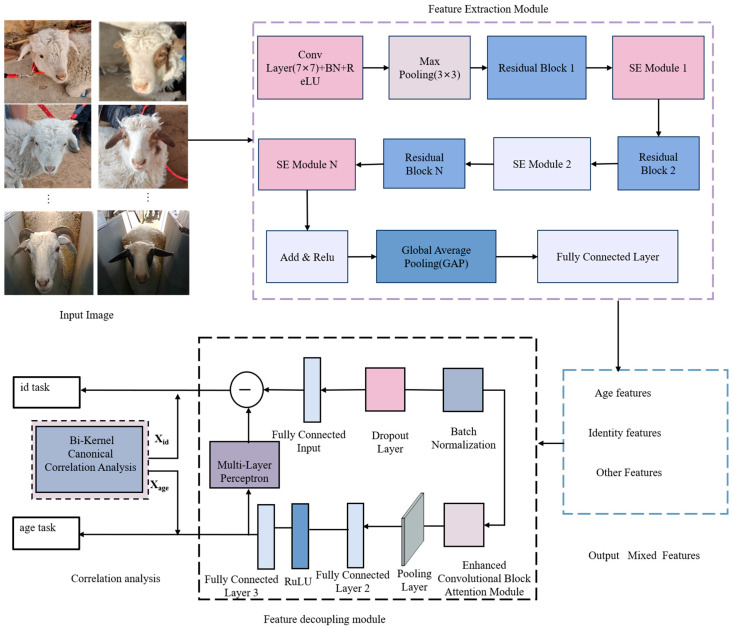
Overview of LBL-SheepNet. The feature extraction network (FE-Net50) feeds into the nonlinear feature decoupling module (ECBAM), followed by the correlation analysis module (BKCCA), and finally the multi-task training module. Acronyms: SE—Squeeze-and-Excitation, ECBAM—Efficient Channel-Attention Module, BKCCA—Bayesian Kernel Canonical Correlation Analysis.

Section 2.3 presents the LBL-SheepNet architecture, which comprises four tightly coupled modules: (1) LBL-SheepNet Model Architecture Overview (Section 2.3.1), (2) a feature-extraction backbone (Section 2.3.2), (3) a nonlinear age-identity decoupling block (Section 2.3.3), (4) a correlation-analysis layer (Section 2.3.4), and (5) a multitask training framework (Section 2.3.5). Each module is detailed in the subsequent subsections, and their joint operation is illustrated later in Figure 3.

#### 2.3.1. LBL-SheepNet Model

In this study, the feature extraction module is utilized as the feature extraction network. The nonlinear feature decoupling module is employed to jointly decompose the age feature vector *X_age_* and the identity feature vector *X_id_*. The correlation analysis module is then utilized to reduce the influence of age-related features on identity information. Finally, the multi-task training module is employed to search for the optimal solution, thereby achieving lifelong biometric learning for sheep face recognition. The entire framework is named the LBL-SheepNet, as illustrated in Figure 3.

#### 2.3.2. Feature Extraction Network

**Figure 4:**
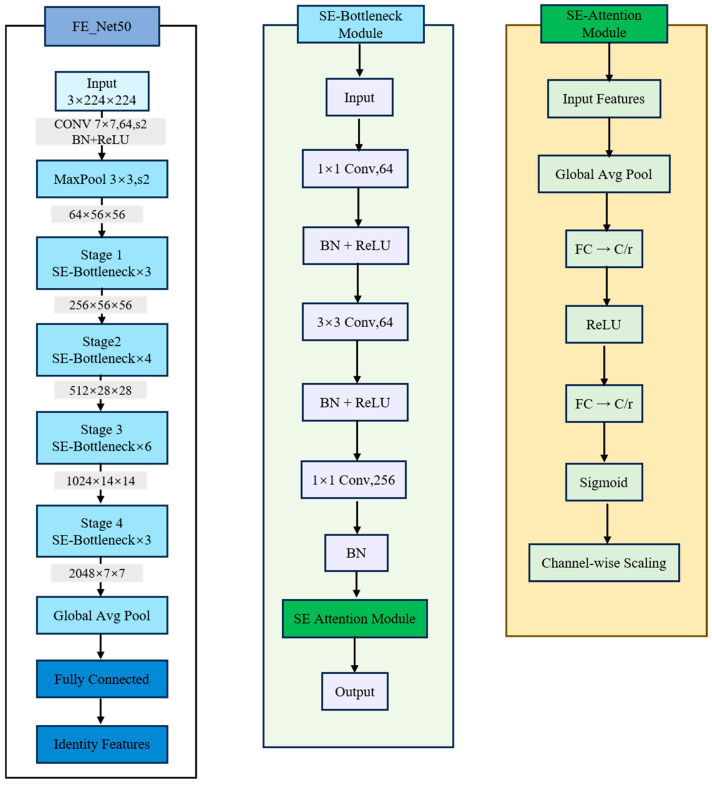
Optimized architectural diagram of the feature extraction network. On the left, the overall process is depicted, where the original framework is enhanced by the introduction of Squeeze-and-Excitation (SE) bottleneck modules to bolster feature extraction capabilities while preserving residual connections. Subsequently, 1 × 1 and 3 × 3 convolutional operations along with attention mechanisms are incorporated into Stages 3 and 4 to refine the feature extraction process.

In the exploration of lifelong biometric learning for sheep face recognition, there has been a notable absence of prior studies focusing on this specific area. Consequently, this research has chosen the representative ResNet50 network model [26] as the foundational framework for our experiments. On the basis of the ResNet-50 network, this study first introduces bottleneck tiny network kernel (BTNK) modules. These modules enhance the model’s ability to capture key features by adding feature extraction layers and attention mechanisms while retaining residual connections. Stage 1 employs 64 filters to extract low-level edges, whereas Stage 2 doubles the channel width to 128 to capture mid-level texture features. Next, 1 × 1 and 3 × 3 convolutional operations and attention mechanisms are added in Stages 3 and 4 to optimize feature extraction and improve the model’s capacity to represent complex features. Finally, a feature fusion module integrates features from different stages using multiple convolutional layers, ensuring feature integrity and effectiveness, and thereby enhancing the model’s accuracy and robustness in handling complex tasks; the structure of the residual network is shown in Figure 4.

Based on the needs of the task, ResNet50 has been improved, where the input image initially passes through a 7 × 7 convolutional layer, followed by a batch normalization and ReLU activation function, and then a 3 × 3 maximal pooling layer, which rapidly reduces the image size from 224 × 224 pixels. As the network progresses, the size of the feature maps gradually decreases while the number of filters increases correspondingly, scaling from 64 to 2048, indicating the network’s progression from learning simple to complex feature representations. At the end of each stage, changes in the dimensions and channel numbers of the feature maps occur; through residual connections and ReLU activation, the network is capable of effectively capturing the hierarchical structural features of the images [27].

The improved residual network focuses on learning global features, and although it is sensitive to changes in sheep facial pose, its fixed-size convolution kernel may not be able to adequately capture the local detailed features of sheep faces. Importantly, the additional parameters introduced by our SE blocks and decoupling modules are supervised by task-specific losses (identity classification and age regression), ensuring that increased model complexity translates into meaningful, age-invariant characteristics, and ensuring that the additional capacity is directed toward learning discriminative, age-invariant features rather than merely expanding the parameter space. Furthermore, its adaptability to local facial pose variations is limited. The complexity of backgrounds can lead to feature confusion in the model, resulting in insufficient feature generalization capabilities [28]. As shown in Table 2, we utilize ResNet50 as the base architecture and introduce the Squeeze-and-Excitation (SE) module to construct the feature-enhanced ResNet50 (FE-Net50) backbone. Additionally, to generate feature distributions and extract information encoding, we incorporate a global pooling layer. In this network structure, we combine the ReLU activation function with the sigmoid control mechanism to complete feature recalibration. The architectural changes listed in Table 2 are visualized in Figure 4, where the SE-BTNK blocks mark the precise locations at which channel-wise recalibration is applied within Stages 3 and 4. Considering that the SE mechanism involves modeling the importance of each channel using two fully connected layers, which poses computational challenges due to its high complexity, we simplify the model parameter complexity by adding 1 × 1 fully connected layers on both sides of the ReLU activation function [29].

#### 2.3.3. Nonlinear Sheep Facial Feature Decoupling Module

In the field of lifelong biometric learning for facial recognition, feature decoupling-based models are primarily categorized into two approaches: linear decoupling and nonlinear decoupling. Linear decoupling is easier to understand than nonlinear decoupling, which involves linearly separating deep facial features from a feature extraction network to obtain identity features consistent across ages [30,31,32,33]. Shakeel et al. [28] and Liu [32] suggest that linear decoupling subtracts identity features obtained through multitask learning with age-restricted features to produce age-independent identity features; however, this ignores that certain features benefit from both growth and identity recognition tasks. Direct subtraction may result in the loss of features beneficial for identity recognition. Furthermore, for deep sheep facial features, relying only on a superficial fully connected layer or the residual mapping module R (∙) (essentially two fully connected layers) does not adequately capture more comprehensive and accurate age features through age-related tasks. In contrast, the nonlinear decoupling model nonlinearly decomposes the sheep facial features extracted by the feature extraction network into identity features and age features. Therefore, this paper proposes a nonlinear feature decoupling module based on an improved attention mechanism combined with an MLP (multilayer perceptron) capable of fitting arbitrary functions for deep decoupling sheep facial features.

**Figure 5:**
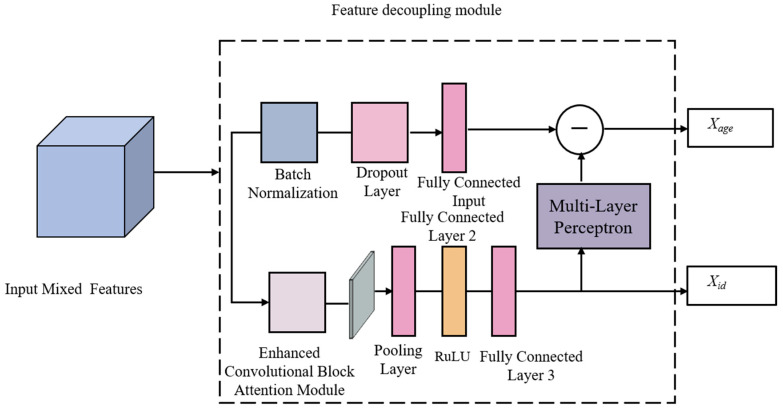
The improved module of feature factorization. Inside the dashed box is an improvement module for the feature decomposition network.

The nonlinear sheep facial feature decoupling module can separate the age feature *X_age_* and identity feature *X_id_* through the joint supervised learning of age-related and identity tasks. To minimize the loss of identity features when separating growth features from sheep facial features, an ECBAM (efficient convolutional attention module) for deep nonlinear sheep facial feature decoupling will be used in this section, as shown in Figure 5.

**Figure 6:**
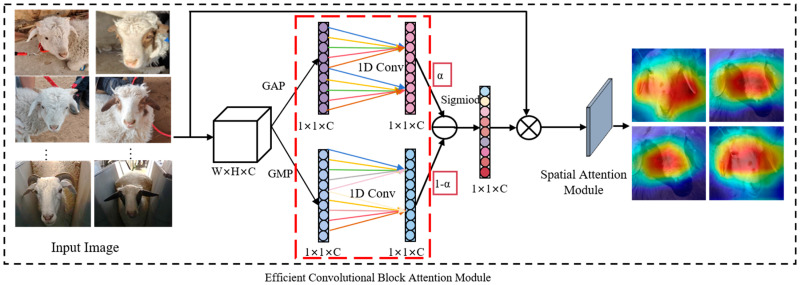
Improved feature enhancement module. The red dotted box shows the area of improvement.

The ECBAM is a pivotal component of the LBL-SheepNet model. Figure 6 illustrates how the ECBAM is integrated into the broader network architecture. Specifically, it receives input from the preceding feature extraction stage and provides support for subsequent nonlinear feature decoupling and correlation analysis. ECBAM performs supervised learning on the growth subnet to enhance the growth features while suppressing other redundant features to obtain more comprehensive and accurate growth features. Then, the MLP is utilized to subtract the identity factor from the extracted growth factor to achieve nonlinear sheep facial feature decoupling and obtain identity features without age-related information, thus eliminating the influence of the growth factor on identity recognition. The architecture shown in Figure 6 is the expanded view of the ECBAM that appears in the dashed box of Figure 5. The CBAM (convolutional block attention module) sequentially utilizes the CAM (channel attention module) and Spatial Attention Module (SAM) to enhance the feature representation capability [34], as shown in Figure 6. In the CAM module, the features after (GAP) global average pooling and GMP (global maximum pooling) are input into the MLP for inter-channel information interaction. However, the compression and expansion operations of the MLP in the CAM module may result in partial information loss. ECA-Net [35] confirms that proper cross-channel interactions can improve the effectiveness of feature extraction in the channel attention module. Therefore, in this paper, ECBAM, which consists of the ECAM (Efficient Channel-Attention Module) and the SAM in tandem, is adopted. As shown in Figure 6, it introduces the trainable parameter α to weigh different channel features. ECAM employs two one-dimensional convolutions, instead of the shared MLP in CAM, to achieve inter-channel information interactions between the GAP and GMP pooled channel features. The convolutional kernel parameters of the two channels are not shared. To enhance the expressiveness of the model, a trainable parameter α is introduced to weigh and sum the information from the two channels. Trained by backpropagation, the model can autonomously select compelling features from the channels. Finally, the sigmoid function is utilized to obtain the weights of each channel.

#### 2.3.4. Correlation Analysis Module

In the LBL-SheepNet model presented in Section 2.2.2, growth features are separated from identity features by incorporating a nonlinear feature decoupling module. This captures the facial features of sheep over time and the unique features used to distinguish different individuals. Subsequently, the introduction of the correlation analysis module quantifies and understands the relationships between different features, particularly identifying potential correlations between growth and identity features. Through correlation analysis, the model can adjust the weights of growth features on identity features via a loss function, thereby improving the model’s accuracy in recognizing individual sheep throughout their lifespan. In addition, correlation analysis can help the model identify and strengthen the features that are more stable and discriminative for individual recognition, further improving the robustness and accuracy of the recognition system. DAL (deep active learning) was proposed by Wang et al. [36], which utilizes the idea of CCA (canonical correlation analysis) and introduces a suitable method for deep learning called BCCA (Bayesian typical correlation analysis). BCCA is implemented through a linear transformation to map high-dimensional features such as growth and identity to a low-dimensional space for correlation analysis. However, it ignores the nonlinear relationships between elements in the feature vectors. Therefore, the kernel approach maps the data to a high-dimensional space by CCA, Bayesian statistics, and MLP methods to identify correlations between variables. The Bayesian framework is used for uncertainty quantification and model selection, leading to the proposed BKCCA (Bayesian kernel typical correlation analysis). The module takes the identity feature vector *X_id_*, age feature vector *X_age_*, and the deviation of their neurons as input. These inputs are then processed by a multilayer perceptron for correlation analysis.

#### 2.3.5. Multi-Task Training of the Model

The three modules jointly perform supervised learning for the identity, growth, and correlation constraints tasks. For identity task discrimination, CosFace loss is used to supervise the learning of identity features *X_id_*. This loss function projects the classification boundary into the cosine angle space, aiming to increase inter-class distance and reduce intraclass distance [19]. The expression for the CosFace loss function is given by Equation (2).

(2) $$L_{id}=\frac{1}{N}\sum_{i=1}-\log\frac{e^{s[\cos\left(\theta y_{i},t\right)-b]}}{e^{s[\cos\left(\theta y_{i},t)-b\right]}+\sum_{j\neq y_{i}}e^{s\times \cos(\theta y_{i},t)\prime }}$$

In this equation, *N* is the number of identity individuals; *y_i_* is the identity label of the *i*-th sample; *s*(*s* > 0) is the scaling factor; and *b* is the constant that controls the cosine boundary.

For the age task, age estimation is performed using linear regression, with the root mean square error (RMSE) used as the loss function. The expression is shown in Equation (3).

(3) $$L_{grow}=\frac{1}{2m}-\sum_{i=1}^{m}{\left[F_{age}\left(x_{age}^{i}\right)-Z^{i}\right]}^{2}$$

In this equation, *X^i^_grow_* is the growth feature vector of the *i-th* sample; and *z^i^* is the growth label of the *i*-th sample.

Combining Equations (2) and (3), the joint loss function for the entire network can be designed to account for age, identity, and constraint modules. To achieve this, a weighted loss function that integrates these conditions can be formulated as follows. The expression is shown in Equation (4).

(4) $$\;L\left(w\right)=L_{id}+\alpha \cdot L_{grow}+\beta \cdot L_{\left(id,grow\right)}$$

The term *L_age_* represents the loss function for the growth condition, which measures the difference between the model’s predicted growth value and the expected growth value.

The term *L_id_* represents the loss function for the identity condition, which evaluates the model’s accuracy in identifying identities.

The term *L*_(*id*, *age*)_ represents the loss function for the constraint module, which measures the model’s compliance with the constraint conditions.

*α* and *β* are the corresponding weights used to adjust the contribution of each condition in the overall loss function.

### 2.4. Deployment of Sheep Facial Recognition System on PCs

Given the practical requirements of sheep farms for efficient and accessible model deployment, this paper details the utilization of TensorFlow Serving for model deployment on personal computers. TensorFlow Serving is a flexible and high-performance system for machine learning models. After training, steps like model extraction and packaging can be quite intricate. Deploying via a cloud server can streamline these tasks. TensorFlow Serving also simplifies model release and maintenance, enabling API-based prediction calls. The models used in this paper for algorithm deployment and experiments can adapt to other data types. Clients can invoke the models through gRPC and HTTP. Specifically, gRPC connects to the sheep face recognition model port, while HTTP is for external client calls.

**Figure 7:**
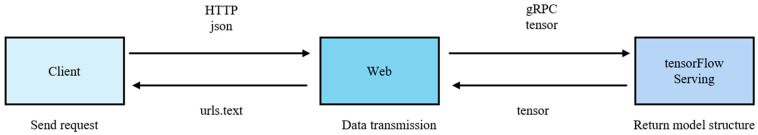
Model deployment flow chart.

Firstly, the LBL-SheepNet model weights are exported. Then, TensorFlow Serving is started and the model path is specified. Subsequently, a web backend program to call the model service is developed. When the frontend sends a request, the web backend receives it, reads the sheep face image data, calls the model service, and returns the prediction results to the frontend. The trained model is exported using the F.Export tool. TensorFlow Serving is initiated to connect the model client program. As TensorFlow Serving demands a fixed folder format and PB file format, the trained sheep face recognition model is converted to PB format using tf.saved_model.simple_save for online deployment. The flowchart is shown in Figure 7.

### 2.5. Experimental Design

#### 2.5.1. Test Environment

The model and tuning process were implemented on a Windows 10 operating system, utilizing an NVIDIA GeForce RTX 3080Ti and PyTorch version 1.7.0 to construct the deep learning algorithm training platform. A batch size of 128 was set, and model optimization was performed using SGD (stochastic gradient descent) [37]. A grid search was performed exclusively on the validation set (5–8 months of age). The search space comprised learning rates {5 × 10^−4^, 1 × 10^−3^, 2 × 10^−3^, 5 × 10^−3^}, momentum values {0.8, 0.9, 0.95}, and weight-decay coefficients {1 × 10^−4^, 5 × 10^−4^}. Each configuration was trained for 100 epochs, and the top-1 identification accuracy on the validation set served as the selection criterion. The optimal hyper-parameters—learning rate 1 × 10^−3^, momentum 0.9, and weight decay 1 × 10^−4^—were fixed for the final model, whose results are reported on the test set that remained unseen during the search.

All comparative evaluations presented in Section 3.2, Section 3.3, Section 3.4 and Section 3.5—including attention-mechanism comparisons, ablation experiments, age-group analyses, and benchmark comparisons with classical architectures—were conducted on the training/validation/test split defined in Section 2.2.1, utilized the hardware and hyper-parameters listed above, and were run once per configuration without additional tuning or data re-partitioning.

#### 2.5.2. Evaluation Protocols

Two complementary splits are employed as follows:

(1) Age-based split (primary protocol, Table 1)

Specifically, 1–4 months for training, 5–8 months for validation, and 9–12 months for testing. The same sheep may appear in multiple windows, but their images are temporally disjoint.

(2) ID-based split (robustness check)

A total of 44 sheep for training, 5 sheep for validation, and 6 sheep for testing; no individual crosses splits.

Experiments not specifically described were conducted in accordance with Protocol 1; the outcomes from Protocol 2 are presented solely in Table 3. Additionally, to further validate the model’s generalization capability in the Discussion section, we performed an additional round of testing using 10 images. These images were captured under varying lighting conditions and included a range of poses. They underwent preprocessing to match the format and quality standards of the existing dataset and were subsequently integrated into the test set. After integration, we reran the model.

#### 2.5.3. Evaluation Indicators

To rigorously assess the performance of the sheep face recognition model, we adopt the following metrics: Precision, Recall, F1-score, IoU (intersection over union), AP@0.5 (average precision at IoU = 0.5), Accuracy, EER (equal error rate), FAR (false acceptance rate), and FRR (False Rejection Rate). All were computed on the test set after hyper-parameters have been frozen using the validation set:

TP (True Positive): the number of correctly identified positive samples.

FP (False Positive): the number of negative samples incorrectly classified as positive.

TN (True Negative): the number of correctly identified negative samples.

FN (False Negative): the number of positive samples incorrectly classified as negative.

From these counts, we derive:

(5) $$Precision=\frac{TP}{TP+FP}$$

(6) $$Recall=\frac{TP}{TP+FN}$$

(7) $$F1-score=\frac{2\times (\mathrm{Precision}\times \mathrm{Recall})}{\mathrm{Precision}+\mathrm{Recall}}$$

(8) $$IoU=\frac{\mathrm{Area}\mathrm{of}\mathrm{Overlap}}{\mathrm{Area}\mathrm{of}\mathrm{Union}}$$

(9) $$mAP@0.5=\frac{\Sigma ᵢAP@0.5ᵢ}{N}$$

(10) $$Accuracy=\frac{TP+TN}{TP+TN+FP+FN}$$

(11) $$EER=\frac{FP+FN}{TP+TN+FP+FN}$$

These metrics provide a comprehensive evaluation of the model’s performance, with higher values indicating better performance for Precision, Recall, F1-score, mAP@0.5, and Accuracy. The lower the error rate (EER), the better the performance.

## 3. Results

### 3.1. Hyperparameter Settings

The weights α and β in Equation (4) were determined by a grid search on the validation set. We varied α ∈ {0.005, 0.01, 0.015, 0.02} while keeping β = 1, and then fixed α = 0.01 while varying β ∈ {0.5, 1}. Each combination was trained for 100 epochs and evaluated by top-1 accuracy on the validation set. The pair (α = 0.01, β = 1) achieved the highest accuracy (95.1%) and was therefore selected for the final model. All numbers in Table 3 are reported on the held-out test set, which was never used during hyper-parameter tuning.

From Table 3, it can be observed that when α is set to 0.01 and β is set to 1, the recognition accuracy is 95.5%, and the mAP@0.5 is 95.3%, which yields the best performance.

**Figure 8:**
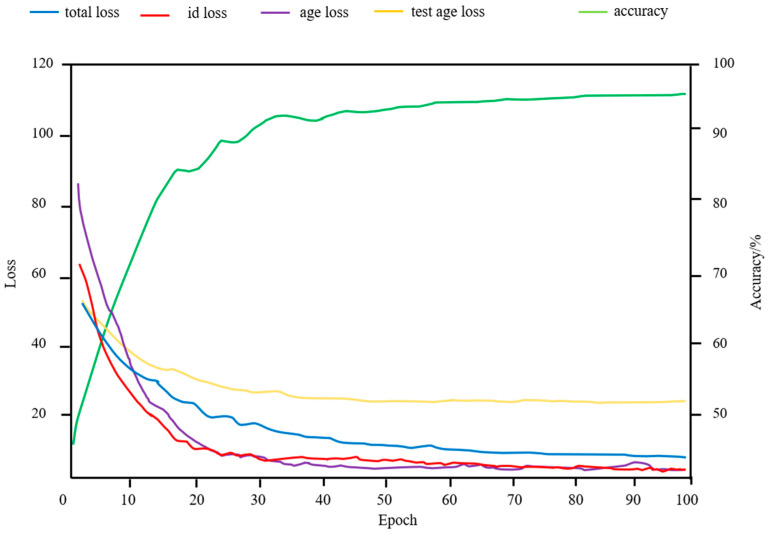
Variation curve of loss value and accuracy during training.

The validation curves when α = 0.01 and β = 1 are shown in Figure 8; the age-loss, identity-loss, and total-loss traces correspond to values computed on the validation set. The validation age-loss converges to a stable plateau.

### 3.2. Comparison of Different Attention Mechanisms

According to the results presented in Table 4, FE_Net50 outperforms the other models in terms of key performance metrics, with a precision of 93.7%, a recall of 92.8%, and a mAP@0.5 of 93.2%. Compared with other attentional mechanisms such as ResNet50+CBAM and ResNet50+CA, although the parameters (26.5 M) and model size (100.0 MB) of the FE_Net50 model are slightly increased, the performance improvement is significant, especially in mAP@0.5. This suggests that the SE unit effectively enhances the feature channel weights, optimizes the feature extraction capability, and thereby improves the model’s performance in sheep face recognition tasks for lifelong biometric learning [38]. Thus, the choice of the FE_Net50 model balances the computational cost with the achievement of higher accuracy and robustness, making it more advantageous for applications that require improved recognition accuracy.

### 3.3. LBL-SheepNet Model Ablation Experiment

Table 5 provides a detailed analysis of the baseline model’s performance following the integration of feature extraction and enhancement modules. The results demonstrate that the inclusion of the feature decoupling module significantly boosts the model’s initial performance. The integration of the SE module initially led to marked improvements in precision, recall, and mAP@0.5, indicating its effectiveness in optimizing the model’s initial performance. The addition of the ECBAM further enhanced the model’s nonlinear feature decoupling capabilities, yielding substantial performance gains, particularly in precision and recall, which increased by 0.5% and 1.1%, respectively. These improvements underscore the ECBAM’s efficacy in managing complex feature decoupling. The combination of the BKCCA with the SE module further refines the model’s performance. BKCCA’s cross-focusing mechanism allows the model to better capture complex correlations between variables, thereby reducing the impact of age-related factors on identification. This in-depth exploration of feature interrelationships enables the model to perform well in more challenging scenarios. The introduction of the MLP module further refines feature decoupling, leading to additional improvements in precision and recall. The MLP not only strengthens nonlinear feature decoupling but also significantly enhances the model’s overall recognition accuracy, especially when used in conjunction with other modules, resulting in substantially improved performance. Optimal performance was achieved by integrating the most effective modules—SE, ECBAM, BKCCA, and MLP. The resulting model demonstrated exceptional metrics, with precision, recall, and mAP@0.5 values reaching 95.5%, 95.4%, and 95.3%, respectively. This suggests that the synergistic effect of combining multiple modules dramatically improves the model’s performance, particularly in tasks involving complex feature decoupling and correlation analysis. A series of ablation experiments confirm that the combination of nonlinear feature decoupling and correlation analysis modules is crucial for enhancing model performance. These findings provide strong empirical evidence for optimizing deep learning models, especially in sheep facial recognition across lifelong biometric learning, a field in which feature decoupling techniques offer distinct advantages.

**Figure 9:**
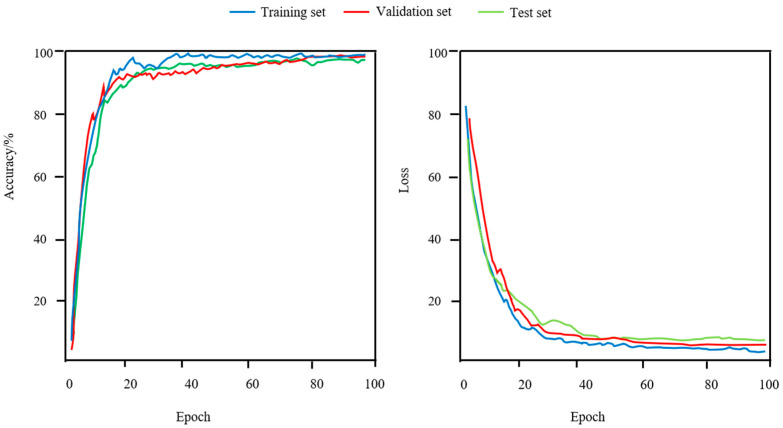
The accuracy and loss curves of the LBL-SheepNet model.

Figure 9 shows the training, validation, and test accuracy and loss trends for the lifelong biometric learning-based sheep face recognition model across iterations. The model rapidly achieves over 90% accuracy, indicating robust generalization. The BKCCA notably enhances handling of age variation by capturing complex correlations. The loss trends demonstrate quick stabilization, with closely tracking curves across datasets, highlighting model stability. The quantitative contribution of BKCCA is demonstrated in Table 5, where its inclusion improves mAP@0.5 by 1.5% relative to the same backbone without BKCCA. The MLP module plays a key role in feature decoupling, improving performance on complex data.

Overall, the integration of enhancement and decoupling modules significantly improves model accuracy and robustness for sheep face recognition through lifelong biometric learning. Additional cross-age evaluation (Section 3.4) and a field test on ten adult sheep under unseen conditions (93.7% accuracy) further confirm generalization beyond the training distribution.

**Figure 10:**
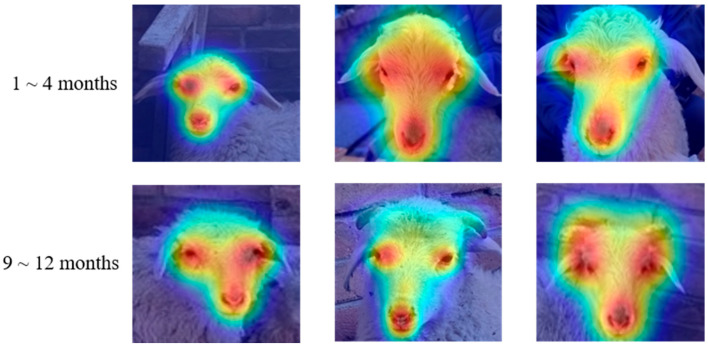
Grad-CAM visualizations of six sheep faces (two per row) showing the model’s attention regions. Warmer colors indicate higher contribution to identity prediction; the eye-nose triangular area is consistently highlighted across different ages, illustrating the focus on age-invariant features.

To evaluate the ResNet50-SFR model, we performed a visual analysis on six test set images using Grad-CAM [39]. As shown in Figure 10, the model’s focus is on the triangular eye and nose area, with the decision region centered between the eyes. Visualizations reveal age -related features primarily between the eyes, emphasizing the need for nonlinear decomposition and correlation analysis modules to separate age and identity factors and minimize growth influence on recognition tasks [40].

### 3.4. Analysis of Recognition Performance of the LBL-SheepNet Model Across Different Age Groups

To evaluate the impact of age on sheep face recognition accuracy, this experiment divided the collected sheep face image dataset, covering ages 1 to 12 months, into four age groups: 1~3 months, 4~6 months, 7~9 months, and 10~12 months. Each age group contained 300 images. Additionally, an independent batch test was conducted on 10 adult sheep, referred to as the field test. This assessment was designed to explore the model’s generalization capabilities beyond the training distribution. The model was trained using the following hyperparameters: a learning rate 0.001, a batch size of 32, and 100 epochs. The model’s performance across different age groups was evaluated using accuracy, precision, recall, and F1-score. Table 6 presents each age group’s accuracy, precision, recall, and F1-scores—additionally, to provide a more intuitive representation of the model’s performance across age groups.

According to the results in Table 6, shows the difference in the performance of the model in different age groups of sheep. The highest accuracy of 95.8% was recorded in the 7~9 month age group which indicates the best performance in this age group. The lower accuracy in the 1~3 month group can be attributed to the immature facial features which hampered the feature extraction. The accuracy of 94.5% in the 10~12 month group was slightly decreased due to the substantial changes in the face, i.e., increased fur coverage which affected the recognition. The 7~9 month group was also ahead in terms of precision and recall, with 95.5% and 96.1%, respectively. However, these metrics were lower for the 10~12 month group, suggesting that the difficulty of recognition increases as facial features evolve. The F1 score of 95.8% for the 7~9 month group indicates strong performance, whereas the lower scores of 94.1% for the 1~3 month group and 94.4% for the 10–12 month group reflect the impact of facial changes on accuracy. Additional cross-age evaluation and a field test on ten adult sheep under unseen conditions (93.7% accuracy) further confirm generalization beyond the training distribution.

The experimental results suggest the model performs best in the 7-month-old to 9-month-old age group. At the same time, the recognition accuracy decreases in the 1-month-old to 3-month-old and 10-month-old to 12-month-old age groups, likely due to significant facial feature changes in these stages. Future research could explore integrating more advanced data augmentation techniques and model optimization strategies to improve recognition performance in these specific age groups and enhance the model’s overall accuracy.

### 3.5. Comparison of EER Metrics from Different Classical Models

To benchmark the proposed LBL-SheepNet, we evaluated six representative architectures—VGG16, VGG19, AlexNet, ResNet50, ViT-Base/16, and our LBL-SheepNet—under identical data partitions (see Section 2.5.2) and hyper-parameter search protocols. All models were trained solely on the training set, tuned on the validation set, and finally assessed on the test set to avoid any data leakage. We employed ViT-Base/16 (86 M parameters, patch 16 × 16) pre-trained on ImageNet-21k; this size was selected for its favorable accuracy–efficiency trade-off on a single RTX 3080 Ti (≈7 GB inference memory) and its 196-token sequence, which balances spatial detail and computational cost for sheep faces. Table 7 summarizes the Equal Error Rate (EER), the primary metric for open-set robustness [39].

LBL-SheepNet achieves the lowest EER (5.7%), outperforming ViT-Base/16 by 1.4 pp and ResNet50 by 5.2 pp. While ViT-Base/16 leverages global self-attention for cross-stage recognition, its patch-wise inductive bias struggles with fine-grained facial details, especially subtle age-invariant cues. In contrast, LBL-SheepNet’s SE-guided channel recalibration and BKCCA-based age–identity decoupling explicitly suppress age-related variance, yielding superior identity robustness.

**Figure 11:**
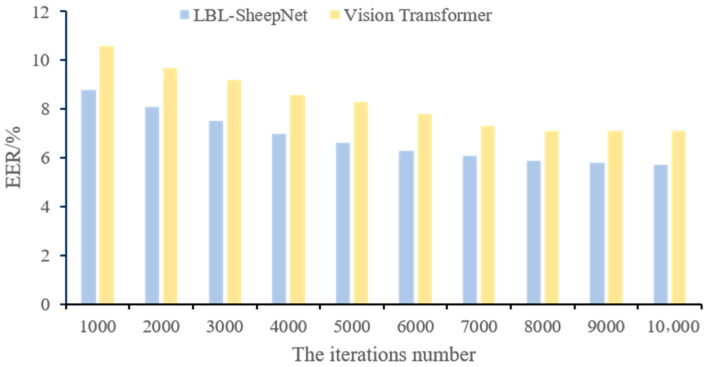
EER of LBL-SheepNet and Vision Transformer under a different number of iterations on the dataset.

To further dissect the training dynamics, we monitored EER across epochs for ViT-Base/16 and LBL-SheepNet, as shown in Figure 11. LBL-SheepNet’s EER decreases monotonically and stabilizes after ~60 epochs, whereas ViT plateaus at 7.1%, confirming the effectiveness of our modular design.

### 3.6. PC-Based Sheep Face Recognition System Testing

**Figure 12:**
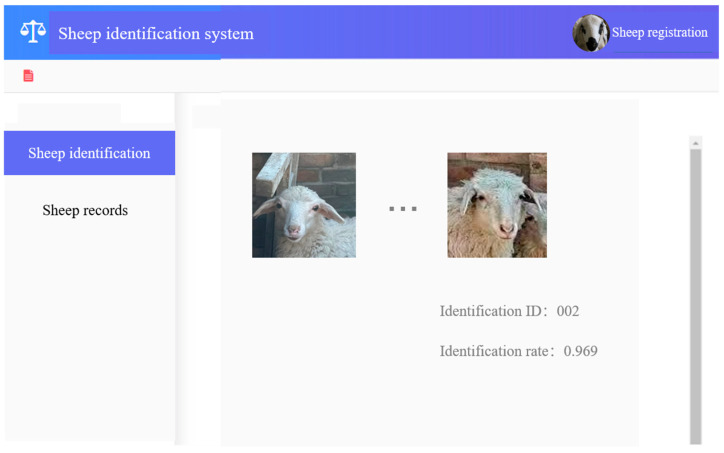
System interface screenshots.

The backend sheep-face recognition program is initiated and the trained model is loaded. Once initialization is complete, the system URL is opened in a browser, and log in is conducted, followed by navigation to the “Sheep Face Recognition” page. Here, the first uploaded image serves as the gallery template (one reference image per sheep), and the second uploaded image serves as the query; identification is successful when the predicted identity matches the corresponding gallery label. Upon clicking on “Upload Image” to load the two local images, “Sheep Face Recognition” is clicked on to display the results within 0.3 s. In the sheep-information query interface, selecting a profile retrieves all records from the database. All code is deployed on a Linux server and has passed full-system testing. The testing results are shown in Figure 12.

## 4. Discussion

Although substantial facial changes occur with age, prior work and the present results demonstrate that identity-discriminative features can still be extracted effectively across different growth stages. This study addresses the critical challenge of maintaining recognition accuracy across varying growth stages, a task at which traditional models falter. Our proposed model stands out through its innovative feature decoupling strategy and adversarial learning approach, which effectively mitigates the impact of age-related changes on identity recognition.

LBL-SheepNet’s primary innovation lies in its ability to separate age-related features from identity-specific ones, allowing for more accurate and stable recognition over time. This is achieved through a multi-module architectural framework that includes a Squeeze-and-Excitation (SE) module, a nonlinear feature decoupling module, and a correlation analysis module with adversarial learning. These components work synergistically to enhance feature representation and reduce age-biased interference. The model’s performance is further validated through a comprehensive evaluation using key metrics such as accuracy, precision, recall, and mAP@0.5, demonstrating its superior performance compared to other state-of-the-art methods like ViT and MobileFaceNet. Due to the fact that facial recognition accuracy hinges on the accurate association of high-confidence detection outcomes with the gallery set, rather than on detection performance per se, this leads to the observed phenomenon where the accuracy surpasses mAP@0.5, reflecting the high confidence in both detection and subsequent identification processes within our controlled test environment.

Despite its strengths, LBL-SheepNet has limitations that warrant further investigation. The model’s performance under conditions of occlusion and varying lighting, common in real-world scenarios, requires more rigorous testing. Additionally, while LBL-SheepNet excels in recognizing known identities, its ability to generalize to completely new sheep needs further validation. Future work should focus on enhancing the model’s robustness against these challenges and improving its generalization capabilities. This includes exploring advanced data augmentation techniques, integrating behavioral or posture data, and developing more resilient model architectures. Although this research primarily focuses on the development and validation of LBL-SheepNet for ovine face recognition across various growth stages, there are several promising directions for future research that could further enhance the model’s performance and expand its application scope. One particularly promising avenue is the exploration of Generative Adversarial Networks (GANs) for age transformation in data synthesis. By adapting GANs to the task of age progression in sheep, we anticipate the potential to synthesize a diverse set of images representing different growth stages. This would enable the model to be trained on a more extensive and varied dataset, thereby improving its robustness and generalization capabilities.

We also acknowledge the limitations of our approach. Although the augmentation strategy is effective, it may not fully capture the diversity of the real world. Future research should explore additional data augmentation techniques or alternative regularization methods to further improve model generalization capabilities. This reassessment highlights the importance of rigorous testing under varied conditions to ensure consistent model performance across different datasets. While the current work focuses on known-herd recognition, the extracted age-invariant features naturally enable open-set extension via cosine similarity thresholds. Future versions will implement full metric learning for unknown sheep-detection.

In practical deployment scenarios, LBL-SheepNet demonstrates competitive accuracy and computational efficiency, making it suitable for edge devices in agricultural settings. Its resilience to environmental variations is commendable, although there is room for improvement in handling occlusion and lighting changes effectively. By comparing LBL-SheepNet with ViT and MobileFaceNet, we highlight its strengths and areas for future enhancement, ensuring that our discussion is aligned with the expectations of the field.

In conclusion, LBL-SheepNet offers a promising approach to lifelong biometric learning for sheep face recognition, addressing key challenges in a practical and innovative manner. The discussions and comparisons provided in this section underscore the model’s strengths and areas for future enhancement, positioning it as a robust solution for smart agricultural platforms.

## 5. Conclusions

This study is dedicated to developing innovative sheep facial recognition technology, focusing on recognition across growth stages, which is of great significance for the intelligent management of large-scale breeding farms. By utilizing non-invasive deep learning techniques, we successfully avoided the potential harm that traditional identification methods might cause to individual sheep. This paper collected facial images of 55 sheep at different growth stages, creating a comprehensive dataset for sheep face recognition across growth stages. We designed and implemented the LBL-SheepNet framework to enhance recognition accuracy, which integrates feature extraction, nonlinear feature decoupling, and correlation analysis modules through a multi-task training strategy. This advanced framework improved the model’s ability to recognize sheep facial features across various age stages. It achieved a recognition accuracy of 95.5% and a mAP@0.5 of 95.3% on the across-growth stages dataset, significantly out-performing traditional methods.

The findings of this research advance the theoretical development of sheep face recognition technology and demonstrate significant practical value in applications. The LBL-SheepNet framework provides an efficient solution for individual sheep identification in large-scale farms, improving automation and intelligent management levels, thereby enhancing production efficiency and economic benefits. We have introduced a new research perspective and practical tools to agricultural technology through this deep learning-driven approach.

## Figures and Tables

**Table 1:** The configuration of the sheep face dataset.

Identity Split	Age Range (Months)	Original Images	Augmentation	Final Images
Training (35 sheep)	1–12	6300	√	25,200
Validation (10 sheep)	1–12	1800	×	1800
Test (10 sheep)	1–12	2000	×	2000
Total	1–12	10,100	/	29,000

**Table 2:** ResNet50 and FE_Net50 model parameters.

Layer	ResNet50	FE_Net50
Conv1	Conv, 7 × 7, 64, stride 2	Conv, 7 × 7, 64, stride 2
Conv2_x	[1 × 1, 64 @ 3 × 3, 64 @ 1 × 1, 256] × 3	[1 × 1, 64 @ 3 × 3, 64 @ 1 × 1, 256] × 3 @ fc[16, 256]
Conv3_x	[1 × 1, 128 @ 3 × 3, 128 @ 1 × 1, 512] × 4	[1 × 1, 128 @ 3 × 3, 128 @ 1 × 1, 512] × 4 @ fc[32, 512]
Conv4_x	[1 × 1, 256 @ 3 × 3, 256 @ 1 × 1, 1024] × 6	[1 × 1, 256 @ 3 × 3, 256 @ 1 × 1, 1024] × 6 @ fc[64, 1024]
Conv5_x	[1 × 1, 512 @ 3 × 3, 512 @ 1 × 1, 2048] × 3	[1 × 1, 512 @ 3 × 3, 512 @ 1 × 1, 2048] × 3 @ fc[128, 2048]
Output Layer	Global average pool, 1000-d fc softmax	Global average pool, 1000-d fc softmax

Note: The @fc[·] notation indicates the SE modules that are inserted after the last bottleneck of each stage, as illustrated by the ‘SE-BTNK’ blocks in Figure 4.

**Table 3:** Recognition rate for different values of α and β.

α	β	Accuracy (%)	mAP@0.5 (%)	Split Used
0.005	1	95.1	94.7	Age-based
0.01	1	95.5	95.3	Age-based
0.015	1	94.8	94.3	Age-based
0.02	1	95.0	94.5	Age-based
0.01	0.5	94.8	94.2	Age-based
0.01	1	94.7	94.1	ID-based

**Table 4:** The comparison results of different attention mechanisms.

Model	Precision (%)	Recall (%)	mAP@0.5 (%)	Parameters (M)	Model Size (MB)	FLOPs (GFLOPs)
ResNet50	91.5	90.6	91.0	25.6	98.5	4.1
ResNet50+SA	92.1	91.7	91.9	26.0	99.2	4.2
ResNet50+CBAM	93.2	92.2	92.9	26.3	99.8	4.3
ResNet50+CA	92.6	91.9	92.3	26.1	99.4	4.2
FE_Net50	93.7	92.8	93.2	26.5	100.0	4.4

**Table 5:** The training results of the ablation experiment.

FE_Net50	CBAM	ECBAM	MLP	BKCCA	Precision (%)	Recall (%)	mAP@0.5 (%)
√					93.7	92.8	93.2
√	√				93.9	93.6	93.5
√		√			94.2	93.9	93.8
√			√		94.3	94.1	93.9
√			√	√	94.6	94.6	93.7
√	√		√	√	95.1	94.7	94.2
√		√	√	√	95.5	95.4	95.3

**Table 6:** Performance Metrics by Age Group.

Age Group (Months)	Text Images	Accuracy (%)	Precision (%)	Recall (%)	F1 Score (%)
1~3	300	94.2	93.8	94.5	94.1
4~6	300	95.1	94.7	95.3	95.0
7~9	300	96.3	95.5	96.1	95.8
10~12	300	95.4	94.0	94.7	94.4
Field Test	200	93.7	93.2	94.0	93.5

Field-test images were collected from 10 adult sheep under novel lighting and postures, not used in training or validation.

**Table 7:** Comparison of Equal Error Rate Metrics for Classical Models.

Model	EER (%)
VGG16	14.3
VGG19	13.0
ResNet50	10.9
AlexNet	11.7
ViT-Base/16	7.1
LBL-SheepNet	5.7

## Data Availability

The original contributions presented in this study are included in the article. Further inquiries can be directed to the corresponding author.
